# OxyR Positively Influences Phaseolotoxin Synthesis and Pyoverdin Production in *Pseudomonas savastanoi* pv. *phaseolicola* NPS3121

**DOI:** 10.3390/microorganisms10112123

**Published:** 2022-10-27

**Authors:** Jackeline Lizzeta Arvizu-Gómez, Alejandro Hernández-Morales, Rafael Arnulfo Juárez-Navarro, Juan Diego Paredes-Tadeo, Juan Campos-Guillén, Juan Ramiro Pacheco-Aguilar, Abril Bernardette Martínez-Rizo, Christian González-Reyes

**Affiliations:** 1 Secretaría de Investigación y Posgrado, Centro Nayarita de Innovación y Transferencia de Tecnología (CENITT), Universidad Autónoma de Nayarit, Tepic 63000, Mexico; 2 Facultad de Estudios Profesionales Zona Huasteca, Universidad Autónoma de San Luis Potosí, San Luis Potosí 79060, Mexico; 3 Unidad Académica de Ciencias Químico Biológico y Farmacéuticas, Universidad Autónoma de Nayarit, Tepic 63000, Mexico; 4 Facultad de Química, Universidad Autónoma de Querétaro, Santiago de Querétaro 76010, Mexico; 5 Unidad Académica de Medicina, Universidad Autónoma de Nayarit, Tepic 63000, Mexico

**Keywords:** *P. savastanoi* pv. *phaseolicola*, OxyR regulon, oxidative stress, phaseolotoxin, pyoverdine

## Abstract

Phaseolotoxin is a major virulence factor of the bean pathogen bacterium *P. savastanoi* pv. *phaseolicola*. This toxin plays a key role in the development of the halo blight disease in bean plants. So far, the signal transduction pathways involved in the synthesis of phaseolotoxin have not been elucidated. The influence of regulation mechanisms related to the oxidative stress response, in particular the OxyR protein, it has been suggested to be involved in this process.. In this study we evaluated the role of OxyR in *P. savastanoi* pv. *phaseolicola*, mainly compared to the synthesis of phaseolotoxin and the virulence of this phytopathogen. Generation of the *oxyR-mutant*, pathogenicity and virulence tests, and analyses of gene expression by RT-PCR assays were performed. The results showed that OxyR exerts an effect on the synthesis of phaseolotoxin and positively influences the expression of the Pht and Pbo cluster genes. Likewise, OxyR influences the production of pyoverdine by the control of the expression of the genes encoding the PvdS sigma factor, involved in the synthesis of this pigment. This study is the first report on members of the OxyR regulon of *P. savastanoi* pv. *phaseolicola* NPS3121.

## 1. Introduction

*Pseudomonas savastanoi* pv. *phaseolicola* (syn *P. syringae* pv. *phaseolicola*; *P. amygdali* pv. *phaseolicola*) is the causal agent of the common bean (*Phaseolus vulgaris* L.) disease known as “halo blight”. It affects leaves and pods, being one of the most destructive diseases of bean cultivation, causing losses of production in the order of 23 to 43%, mainly in temperate or cold areas [1,2]. The disease is characterized by the presence of chlorotic areas around the necrotic site of infection. In cases of severe infection, the plants develop a systemic generalized chlorosis presenting growth deficiencies, with wrinkled and mottled upper leaves [1,3]. Among the virulence factors that *P. savastanoi* pv. *phaseolicola* synthesizes for the development of the disease, is the phytotoxin known as phaseolotoxin, which is the main factor responsible for the characteristic chlorotic symptoms of the halo blight [4,5,6]. The phaseolotoxin [Nδ (N’-sulfodiaminophosphinyl)-ornithyl-alanyl-homoarginine] is an extracellular toxin, non-host-specific, which is produced by the bacteria within the site of infection and diffuses into leaf tissues causing chlorophyll synthesis inhibition [5,7,8]. In addition, phaseolotoxin facilitates the systemic invasion of the plant, contributing significantly to the virulence of the pathogen. Phaseolotoxin is then a key element for the development of the disease and an important virulence factor of the bacterium [5,6,9]. The production of phaseolotoxin by *P. savastanoi* pv. *phaseolicola* is regulated by the temperature, with optimal production at 18–20 °C, while at 28 °C (the optimal growth temperature for this bacterium) it is not detected [10,11].

Genes required for phaseolotoxin synthesis and, therefore, for the virulence of the bacterium are located within a 30.24 kb horizontally acquired pathogenicity island name “Pht cluster”, where they are organized in five transcriptional units including two monocistronic (*argK* and *phtL*) and three polycistronic operons, one comprising of 11 genes from *phtA* to *phtK* with an internal promoter driving expression of *phtD* to *phtK* and a third large polycistronic operon comprising of 10 genes from *phtM* to *phtV* [12,13,14]. Recent studies have identified and characterized a second chromosomal region of *P. savastanoi* pv. *phaseolicola* NPS3121 called as “Pbo cluster”, which is also involved in the phaseolotoxin synthesis. The Pbo cluster contains 16 open reading frames arranged in four transcriptional units, including one monocistronic (*pboJ)* and three polycistronic (*pboA-pboI, pboK-pboN,* and *pboO-pboP*) [15]. Analyses of gene expression of the Pht and Pbo clusters have shown thermoregulation of the phaseolotoxin genes, whose expression occurs selectively at low temperatures (18 °C) with high levels of transcripts and increase of the promoter activity of the most of these genes under this condition, with exception of the *pboJ* gene (Pbo cluster), is constitutively expressed at both 18 °C and 28 °C [12,15].

The mechanisms that control the gene expression of phaseolotoxin in *P. savastanoi* pv. *phaseolicola* are just beginning to be elucidated. However, the advances that have been made in this regard have shown that the regulation of the Pht and Pbo clusters is complex and involves many factors, including both specific regulatory proteins encoded within the Pht cluster itself (PhtL, PhtA, PhtABC) [12,16,17,18] and global regulators (IHF, the two-component GacS-GacA system and a putative 14–20 kDa regulator protein) [19,20,21]. Most of these factors appear to mediate the regulation of genes of the Pht and Pbo clusters in response to low temperature. 

Transcriptomics analyses, comparing the gene expression profile of the bacterium *P. savastanoi* pv. *phaseolicola* NP3121 grown at 18 °C and 28 °C (corresponding to related conditions and not with the phaseolotoxin synthesis, respectively) showed that, at low temperatures (18 °C), the overall expression profile obtained for *P. savastanoi* is like that reported for *Pseudomonas aeruginosa* after exposure to the oxidative stress inducing agent-hydrogen peroxide, resulting in an expression pattern of oxidative stress response [22]. Therefore, these analyses suggested that low temperatures (18 °C) induce oxidative stress in the *P. savastanoi* pv. *phaseolicola* cells, which in turn, give rise to the gene expression profile obtained, among which are highlighted the expression of the Pht and Pbo cluster phaseolotoxin genes [22]. Recent studies of our work group showed that the gene expression of the Pht cluster is regulated by oxidative stress in a manner dependent of the reactive oxygen species (ROS) present in the cell, thereby demonstrating that the expression of the Pht cluster genes is part of the oxidative stress response in *P. savastanoi* pv. *phaseolicola* NPS3121 [23]. Moreover, these results suggest possible regulatory pathways involved in the phaseolotoxin synthesis, where the regulation mechanisms of oxidative stress response could be involved. 

The OxyR regulon has been identified and characterized in the *E. coli* model bacterium as one of the main regulatory pathways for the response to oxidative stress, which responds, in particular, to the existence of hydrogen peroxide (H_2_O_2_) in the cells. The regulatory action of OxyR is mediated by the activation of the protein, directly, through the oxidation of the disulphide bonds by H_2_O_2_. Once activated, OxyR induces the expression of genes within the OxyR regulon, which mainly protects the cell from H_2_O_2_ toxicity [24,25,26]. The OxyR dependent regulation of primary antioxidant genes is conserved in a variety of bacteria. However, recent investigations have revealed differences in the members of the OxyR regulon among bacteria [27,28,29]. Furthermore, it has been demonstrated that there is diversity in the activation mechanisms of OxyR orthologs in other microorganisms [30]. Studies in diverse phytopathogenic bacteria have demonstrated that OxyR participates in the synthesis of some pathogenicity and/ or virulence factors, but this influence is variable or specific for the phytopathogen [31,32,33]. Although OxyR is primarily thought of as a transcriptional activator, in some bacteria it can function as either a repressor or an activator under both oxidizing and reducing conditions [30]. Thus far, the role of the OxyR global regulator in *P. savastanoi* pv. *phaseolicola* NPS3121 has not been evaluated and no member of the OxyR regulon in this phytopathogen has been reported. Therefore, this study was undertaken with the objective to evaluate the influence of OxyR in the production of virulence determinants in *P. savastanoi* pv. *phaseolicola* NPS3121, mostly focused on the synthesis of phaseolotoxin. The present work demonstrates that the OxyR global regulator positively influences the synthesis and gene expression related to phaseolotoxin, contributing to the knowledge about the regulatory pathways involved in this process. Additionally, based on the results obtained, this work demonstrates that like phaseolotoxin, synthesis of pyoverdine is also a member of the OxyR regulon of *P. savastanoi* pv. *phaseolicola* NPS3121. 

## 2. Materials and Methods

### 2.1. Bacterial Strains, Media, and Growth Conditions

The *Pseudomonas savastanoi* strains: pv. *phaseolicola* NPS3121 wild type [34], *oxyR-mutant*, *oxyR-* complemented mutant (*oxyR- C*) and *P. syringae* pv. *tomato* DC3000 were grown or maintained on King´s B (KB, Sigma–Aldrich^®^, MO, USA) medium or in M9 minimal medium (Sigma–Aldrich^®^, MO, USA) at 18 °C or 28 °C. The *Escherichia coli* strains were routinely grown on Luria–Bertani (LB, Dibico^®^, EdoMex, Mexico) medium at 37 °C. The *E. coli* Top 10 strain (Invitrogen, CA, USA) was used in the cloning procedures. The *E. coli* strain JM103 [35] was used as an indicator strain in the phaseolotoxin assays. When required, the following antibiotics were added for the *P. savastanoi* strains and *E. coli*, respectively: ampicillin (Amp) (300 μg mL^−1^, 100 μg mL^−1^), kanamycin (Km) (70 μg mL^−1^, 50 μg mL^−1^), gentamicin (Gm) 30 μg mL^−1^, and rifampin (Rf) 50 μg mL^−1^.

### 2.2. Molecular Biology Techniques

Genomic DNA of *P. savastanoi* pv. *phaseolicola* NPS3121 was isolated as previously described [36]. Routine molecular techniques were performed using standard protocols [37]. Plasmids were purified using the GenElute^TM^ Plasmid Miniprep Kit (Sigma-Aldrich^®^, St. Louis, MO, USA). Restriction enzymes were used according to instructions provided by the suppliers. The PCR products were amplified with *Taq* DNA Polymerase (Thermo Fisher Scientific, Waltham, MA, USA). Primers were designed using the Vector NTI Software (Invitrogen, Carlsbad, CA, USA) based on the reference genome of *P. savastanoi* pv. *phaseolicola* 1448A (Gene Bank accession no. CP000058) [38]. The oligonucleotides used in this study are listed in Table 1. 

### 2.3. Construction of the P. savastanoi pv. phaseolicola NPS3121 oxyR- Mutant Strain and oxyR- Complemented Mutant (oxyR- C) 

The generation of the *oxyR-mutant* of *P. savastanoi* pv. *phaseolicola* NPS3121 was carried out through the mutational strategy of gene inactivation by the integration of a homologous suicide plasmid, based on the methodology previously described [40]. An internal fragment (493 bp) of the unique homologous gene *oxyR* (PSPPH_RS00995; old_locus_tag PSPPH_0190; 924 bp) was obtained by PCR using the primers pairs OxyR-FWD:AGCAGGCTCAAGGTATTCGT and OxyR-REV:GAAGCGACCATGTGCCGAAT (Table 1). The PCR amplification was performed at 94 °C for 3 min for 1 cycle; 94 °C for 35 s, 55 °C for 35 s, and 72 °C for 1 min for 35 cycles; and 72 °C for 7 min for 1 cycle. The amplicon was cloned into the vector pCR4-TOPO (Invitrogen, CA, USA) obtaining the recombinant molecule pCR4-TOPO::*oxyR* disrupted, in which the orientation and validation of the insert was carried out by restriction and sequencing analyses. This recombinant molecule (pCR4-TOPO::*oxyR* disrupted) was incorporated into the working strain by electroporation using the following parameters: 200 V, 20 Ω y 250 µFD. The obtained transformants were selected in KBRfKmAmp medium. Finally, the cointegration of the suicide plasmid in the target region was confirmed by PCR. The complementation in trans of the *oxyR-mutant* strain was performed by cloning of the *oxyR* gene (PSPPH_RS00995) in the pBBR1MCS-5 plasmid [41]. A DNA fragment containing the *oxyR* open reading frame and 250 bp upstream from the ATG codon was obtained by PCR using the pOxyR250-*Sma*I-OxyRRev-*Bam*HI oligonucleotides (Table 1). The PCR amplicon was digested by *Sma*I-*Bam*HI restriction enzymes and ligated into the pBBR1MCS-5 plasmid digested with the same enzymes. The resulting plasmid pBBR1MCS-5::*oxyR250* was introduced by electroporation in the *P. savastanoi* pv. *phaseolicola oxyR-mutant* strain. 

### 2.4. Bacterial Growth Curves of the P. savastanoi pv. phaseolicola NPS3121 Strains

The growth dynamics of the strains: wt, *oxyR-mutant*, and *oxyR- C* (complemented mutant) of *P. savastanoi* pv. *phaseolicola* NPS3121, were evaluated in both M9 minimal medium (MM9)(2X) supplemented with 0.4 mM CaCl_2_, 4 mM MgSO_4_ and glucose (0.8%) and King´s B (KB) media at 18 °C and 28 °C. The bacterial growth curves in MM9 were carried as follows: pre-inoculums (30 mL) of the wt, *oxyR-mutant*, and *oxyR- C* strains, were grown in MM9 at 28 °C for 24–48 h. The cells were inoculated into 100 mL of MM9 at optical density (OD_600nm_) 0.1. To evaluate the effect of the temperature, the cultures were incubated at 18 °C or 28 °C. On the other hand, the growth conditions in the KB rich medium consisted of: pre-inoculums (30 mL) of the strains wt, *oxyR-mutant*, and *oxyR- C*, were grown in KBRf, KBRfKmAmp, and KBRfKmAmpGm broth, respectively, at 28 °C for 24 h. These pre-inoculums were used to inoculate 100 mL of KB broth at optical density (OD_600nm_) 0.05. The cultures were incubated at 18 °C or 28 °C. Two biological replicates with two technical replicates were evaluated.

### 2.5. Bacterial Growth Conditions and RNA Isolation

The growth conditions used for the expression analyses corresponded to those previously used in studies of genic expression of virulence (phaseolotoxin) and pathogenicity factors [12,22,23]. *Pseudomonas savastanoi* pv. *phaseolicola* NPS3121 strains were grown at 18 °C and 28 °C in M9 minimal medium (MM9) (2X) supplemented with 0.4 mM CaCl_2_, 4 mM MgSO_4_ and glucose (0.8%) (Golden-Bell Reactivos^®^, Jal, Mexico) as the carbon source. The growth conditions were as follows: pre-inoculums (30 mL) of the wt and *oxyR-mutant* strains of *P. savastanoi* pv. *phaseolicola* were grown in M9 minimal medium at 28 °C for 24–48 h. The cells were inoculated into 100 mL of MM9 at optical density (OD_600nm_) 0.1. The cultures were incubated at 18 °C or 28 °C and grown until they reached the late log phase, growth stage in which the cells were recovered to further RNA extraction. The cells cultures were distributed in 50 mL conical tubes and recovered by centrifugation (GYROZEN^®^ 1580R, Corea) to 4000 rpm for 15 min at 4 °C. Supernatants of each culture were removed to further assays of phaseolotoxin production. For RNA isolation, the cell pellets were subjected to one washing step with sterile deionized water prior to their storage at −80 °C. Total RNA was extracted using the TRIzol Reagent following the manufacturer´s instructions (Invitrogen, CA, USA). The RNA was eluted in 50 μL of diethylpirocarbonate (DEPC)-treated water. Genomic DNA was removed by digestion with DNase I (Invitrogen, USA). The quantity and quality of the RNA was determined using a spectrophotometer (Multiskan-GO, Thermo Fisher Scientific, MA, USA). The integrity of RNA samples was confirmed by analytical agarose gel electrophoresis.

### 2.6. Analysis of Expression by Reverse Transcriptase (RT)-PCR Assays

The gene expression analyses were performed by the RT-PCR technique using the oligonucleotides pairs listed in Table 1. Analyses were performed with two biological replicates using RNA isolated from a different culture and two technical replicates with the same RNA samples. The SuperScript One-Step RT-PCR kit (Invitrogen, CA, USA) was used according to the manufacturer’s instructions with 200 ng RNA template per reaction. The RT reaction was performed at 50 °C for 30 min, followed by PCR amplification at 94 °C for 2 min for 1 cycle; 94 °C for 35 s, 55–60 °C for 40 s, and 72 °C for 1–2 min for 23–28 cycles; and 72 °C for 7 min for 1 cycle. Controls used for each set of primers were: (i) PCR without the reverse transcription step to verify the absence of DNA; (ii) RT-PCRs without RNA templates to detect any contaminating DNA/RNA; (iii) PCRs using chromosomal DNA as template to ensure primer fidelity. Furthermore, the amplification of a portion of the *16S* rRNA operon using suitable primers was used as an internal control of the reaction (23 amplification cycles). Twenty-eight amplification cycles were used in most of the RT-PCR assays. Number cycles in which the differential expression widely reported, for the Pht cluster genes in function of the temperature (28 °C vs. 18 °C) [12,22,23] was observed in these assays.

### 2.7. Phaseolotoxin Bioassays

Phaseolotoxin production by the *P. savastanoi* pv. *phaseolicola oxyR-mutant* strain was evaluated by the *E. coli* JM103 strain growth inhibition assay as previously described [42,43], with some modifications. The supernatants of the cell cultures of the *oxyR-mutant* strain grown at 18 °C and 28 °C in M9 minimal medium were recovered as described above. Aliquots of 100 μL of the supernatants were deposited on sterile 6 mm Whatman paper discs and left until dry to subsequently transfer them onto MM9 agar plates previously seeded with the *E. coli* JM103 control strain. The plates were incubated at 37 °C for 24 h. In every case, plates containing arginine were used as controls to confirm that growth inhibition was because of phaseolotoxin. Simultaneously, cultures of the *P. savastanoi* pv. *phaseolicola* NPS3121 wt strain and *oxyR-* complemented mutant (*oxyR- C*) grown at 18 °C and 28 °C in M9 minimal medium were evaluated and used as a control. 

### 2.8. Pathogenicity and Virulence Assays in Bean Pods

Bacteria were tested for pathogenicity and virulence using the modified bean pod assays previously described [44]. Bacterial cells grown in KB medium agar plates supplemented with the corresponding antibiotics, were removed with a sterile toothpick, and inoculated in detached bean pods previously disinfected with NaClO 5%. For each bacterial strain, three pods were inoculated. The inoculated bean pods were placed inside an airtight container into which these were arranged on a surface positioned about 3–4 cm from a base of paper moistened with sterile water to maintain humidity. The containers were maintained at 28 °C or 18 °C for 3–5 days. As controls, inoculations with cells of *P. syringae* pv. *tomato* DC3000 were included. Likewise, pods treated only with sterile toothpick without bacteria were included as a control. 

### 2.9. Quantification of Siderophores

The *P. savastanoi* pv. *phaseolicola* NPS321 strains: wild type, *oxyR-mutant*, and *oxyR- C*, were grown in M9 minimal medium at 28 °C and 18 °C with shaking at 200 rpm. Cell-free supernatants were obtained by centrifugation at 4000 rpm for 15 min at 4 °C and filtered through a syringe filter (0.22 μm). The supernatants were analysed at 400 nm to determine pyoverdine production. The pyoverdine concentration was calculated using the molar absorption coefficient (ε = 20000 M^−1^ cm^−1^) [45]. The values were normalized with the biomass of the cultures at 600 nm and expressed in µM of pyoverdine [46]. Three biological replicates were evaluated. Statistical analysis was performed using the statistical software GraphPad Prism Version 5.01. Analysis of variance (ANOVA) was performed with Tukey´s method to compare the means between treatments (*p* < 0.05).

## 3. Results

### 3.1. OxyR Positively Influences on the Growth Rate of P. savastanoi pv. phaseolicola NPS3121

**Figure 1:**
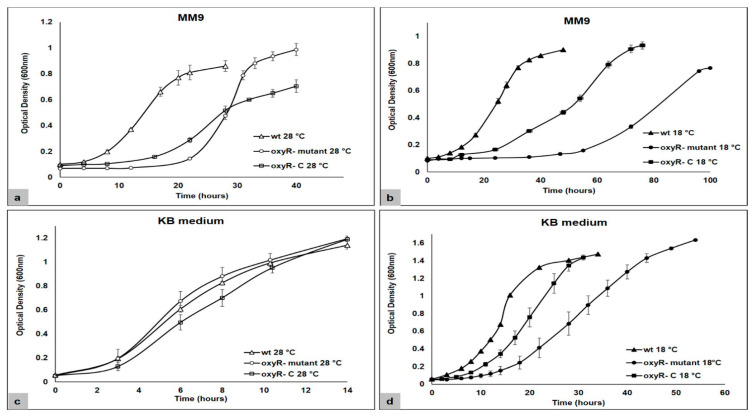
Growth curves of the wt, *oxyR-mutant*, and *oxyR- C* (complemented mutant) strains of *P. savastanoi* pv. *phaseolicola* NPS3121. (**a**) Bacterial growth curves at 28 °C in MM9. (**b**) Bacterial growth curves at 18 °C in MM9. (**c**) Bacterial growth curves at 28 °C in KB medium. (**d**) Bacterial growth curves at 18 °C in KB medium.

To evaluate the role of OxyR on the physiology of the *P. savastanoi* pv. *phaseolicola* bacterium, specifically in relation to the production of virulence determinants, we first constructed the *oxyR-mutant* in *P. savastanoi* pv. *phaseolicola* NPS3121. Obtaining the *oxyR-mutant* was achieved by the integration of a homologous suicide plasmid in the unique homologous gene *oxyR* (PSPPH_RS00995; PSPPH_0190). The integration of the suicide plasmid in the interest target was validated by PCR assays [47]. Because the pathogenicity and virulence capacity of various phytopathogenic bacteria is a function of their growth [48,49], we obtained bacterial growth curves of the *oxyR- P. savastanoi* pv. *phaseolicola* NPS3121 mutant strain under conditions related or not (18 °C or 28 °C, respectively) to its virulence or pathogenicity [22]. This aimed to evaluate the influence of OxyR in the growth of *P. savastanoi* pv. *phaseolicola*. First, the results showed that in both the *oxyR-mutant* and the wild type (wt) strain, low temperatures (18 °C) decreased the bacterial growth rate in both M9 minimal medium and King´s B media (Figure 1). On the other hand, the analysis of the growth kinetics in MM9 showed that in lacking functional OxyR, the growth rate of *P. savastanoi* pv. *phaseolicola* was diminished; this at both 18 °C and 28 °C. At 28 °C, the optimal growth temperature for this bacterium, the growth rate in the *oxyR-mutant* decreased approximately 1.5-fold relative to the wt strain. While the comparison of the growth rates among the *P. savastanoi* strains (wtvs. *oxyR-*) at 18 °C showed a decrease of 3-fold in the *oxyR-* relative to the wt strain (Figure 1). This behaviour was reproducible in all performed kinetics. Contrary to what was observed in MM9, the bacterial growth curves in the rich KB medium showed that at 28 °C the lack of OxyR did not influence the growth rate; only at 18 °C was there observed a decrease in the growth rate in the *oxyR-mutant* compared to the wt strain (Figure 1). These results suggest that OxyR is implicated in the bacterial growth process of *P. savastanoi* pv. *phaseolicola* NPS3121. 

To validate these results, the construction and evaluation of the bacterial growth curves of the *oxyR-* complemented mutant strain (*oxyR- C*) was performed. An increase in the bacterial growth rate was observed in the *oxyR- C* strain compared to the *oxyR-mutant* strain in most of the evaluated conditions (Figure 1). The introduction in trans of *oxyR* only partially completed the growth phenotypes. In general, these results demonstrate that OxyR plays an important role in the growth of *P. savastanoi* pv. *phaseolicola* NPS3121, positively influencing this process. This positive influence occurs mainly at low temperatures (18 °C) and under minimal media conditions. On the other hand, because previous work has indicated that in the late log growth phase there occurs expression of virulence genes [22,23], these bacterial growth assays allowed us to establish the growth stage for this study, in particular compared to MM9. Additionally, because the expression and activity of OxyR are dependent on the growth stage, being most active in the log phase and less in the stationary phase [50], these growth curves allowed us to obtain the cell cultures under functionality conditions of OxyR.

### 3.2. The Virulence of P. savastanoi pv. phaseolicola NPS3121 Is Influenced by the OxyR Global Regulator

**Figure 2:**
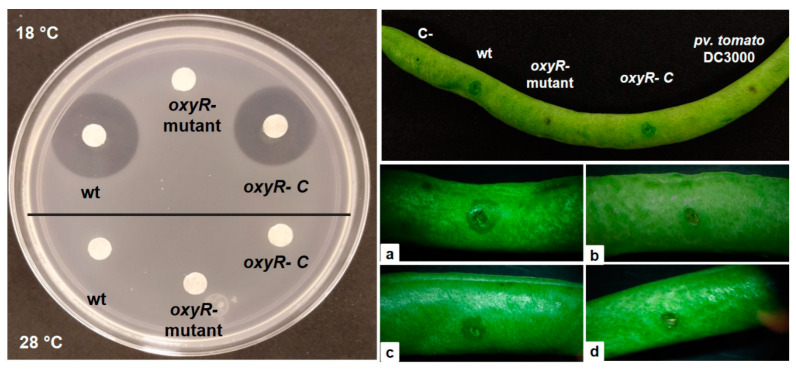
Phaseolotoxin synthesis test and bean pod pathogenicity assays. Left panel shows the phaseolotoxin production assays in vitro. Right panel shows the bean pod assays. (**a**) *P. savastanoi* pv. *phaseolicola* wt strain. (**b**) *oxyR-mutant* strain. (**c**) *oxyR-* complemented mutant strain [*oxyR- C*]. (**d**) *P. syringae* pv. *tomato* DC3000 strain.

As above mentioned, in various bacteria, the OxyR protein influences the virulence and/or pathogenicity of these microorganisms; the influence is variable (positive or negative) [27,31]. To evaluate whether OxyR participates in the virulence of *P. savastanoi* pv. *phaseolicola* NPS3121, we performed in vitro production tests for phaseolotoxin, an important virulence factor of the bacterium, using the *oxyR-mutant* and the *oxyR- C* strains. The results showed the absence of formation of halos of growth inhibition in the *oxyR-mutant* strain assays at both evaluated temperatures (18 °C and 28 °C), compared with the wt strain, which showed the presence of halos of growth inhibition in assays of cultures grown at 18 °C (Figure 2). These halos are representatives of the capacity for phaseolotoxin production by the bacteria. As was expected, the absence of halos of growth inhibition in assays of supernatants of the wt strain grown at 28 °C was observed, congruent with the existent thermoregulation (18 °C) on the synthesis of phaseolotoxin [10,11]. Furthermore, the phaseolotoxin assays showed that the presence of the *oxyR* gene in trans in the *oxyR-mutant* background restores the ability for production of phaseolotoxin in this bacterium at 18 °C (Figure 2).

Additionally, plant inoculation tests were performed by bean pod assays as previously described [44]. The results of the bean pod inoculation tests showed the presence of similar necrotic spots among the strains wt, *oxyR-mutant*, and *oxyR- C*, which is characteristic of the infection process, while only the wt and *oxyR- C* strains were able to produce the characteristic water-soaked lesion related to virulence processes. As the control, we inoculated the *P. syringae* pv. *tomato* DC3000 strain, which elicited the expected hypersensitive response (HR), and inoculum without bacteria (negative control), which did not produce any symptoms, ruling out that the necrotic spot observed was caused by the inoculation procedure (Figure 2). All these results demonstrate that OxyR is necessary to the phaseolotoxin synthesis and in the virulence of *P. savastanoi* pv. *phaseolicola* NPS3121. 

### 3.3. OxyR Positively Influences the Expression of Genes Involved in the Phaseolotoxin Synthesis

**Figure 3:**
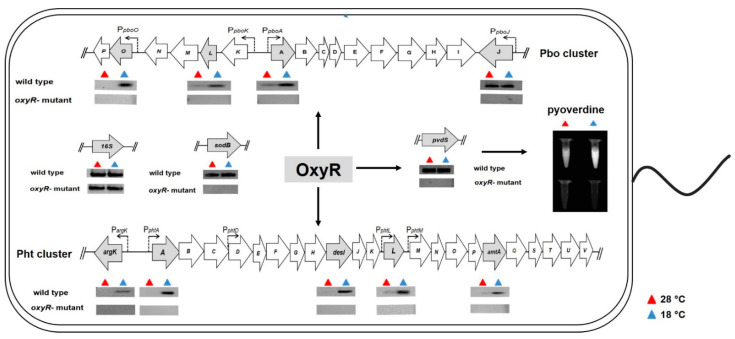
Expression analysis by RT-PCR assays and pyoverdine production. The figure depicts the effects that the OxyR protein exerts on the expression of the studied genes and on the pyoverdine synthesis. In the figure is included a graphic representation of the Pht and Pbo clusters and *sodB, pvdS* genes. The housekeeping *16S rDNA* gene (16S) was also evaluated and used as an internal control. Each arrow represents an individual gene, with the direction of the arrow indicating the direction of transcription. Gray arrows indicate the genes evaluated by RT-PCR analyses, which are shown under each gene evaluated by a band amplified (black) or absent in wild type and *oxyR-mutant* al 28 °C (indicated by red triangle) and 18 °C (indicated by blue triangle). Twenty-eight amplification cycles were used in most of the RT-PCR assays. The *16SrRNA* was evaluated using 23 cycles of amplification. Representative image showing the production of fluorescent siderophore pyoverdine assessed under UV light is also included.

Once it was demonstrated that OxyR influences the synthesis of phaseolotoxin by in vitro phaseolotoxin assays, we decided to evaluate whether this influence is related to processes of regulation and/or expression of phaseolotoxin genes. Currently, the Pht and Pbo cluster genomic regions have been identified as involved in the synthesis of phaseolotoxin in *P. savastanoi* pv. *phaseolicola* [12,15]. The RT-PCR assays were performed to analyse the expression pattern of genes of the Pht and Pbo clusters in the *oxyR-mutant* background grown at both 28 °C and 18 °C. As a control, the wt strain cultures grown in similar conditions were assayed, simultaneously. Five genes (*argK, phtA, desI, phtL*, and *amtA*), representatives of the five transcriptional units that make up the Pht cluster, were selected and evaluated in these assays. An expression pattern dependent on low temperatures (18 °C) of the Pht cluster genes was observed in the wt background, as expected, while for that in the *oxyR-mutant*, no transcripts signal was observed at both 28 °C and 18 °C for all the genes evaluated (Figure 3). These results showed that OxyR positively influences the expression of the Pht cluster genes, particularly at 18 °C. 

Regarding the Pbo cluster genes, RT-PCR analyses were performed by evaluating the expression of four genes (*pboO*, *pboL*, *pboA*, and *pboJ*) representative of the four transcriptional units that make up this region. These assays showed the expression pattern expected for the Pbo cluster genes in the wt background, with higher transcript levels at 18 °C compared to 28 °C, with the exception of the *pboJ* gene, which is similar at both temperatures. The assays in the *oxyR-mutant* background showed the absence of transcripts signals in all the evaluated genes at both temperatures (Figure 3). These results suggest that the OxyR protein participates in the regulation of the expression at low temperatures (thermoregulation) of the phaseolotoxin genes. 

### 3.4. In P. savastanoi pv. phaseolicola NPS3121, the Pyoverdine Synthesis Is Also a Member of the OxyR Regulon

**Figure 4:**
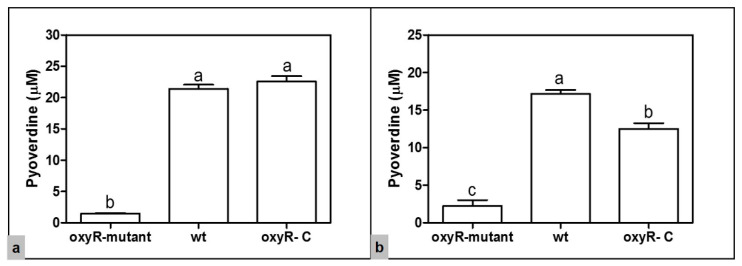
Pyoverdine production by the *P. savastanoi* pv. *phaseolicola* NPS3121 strains. (**a**) Pyoverdine levels in the cell free supernatants of the wt, *oxyR-mutant*, and *oxyR- C* strains, grown at 28 °C. (**b**) Pyoverdine levels in the cell free supernatants of the wt, *oxyR-mutant*, and *oxyR- C* strains, grown at 18 °C. Bars represent the means ± SD (*n* = 3). Different letters represent means that are statistically different (*p* < 0.05).

A phenotypic feature of the cultures of the *oxyR-mutant* strain, observed during the analyses of bacterial characterization (e.g., bacterial growth curves and virulence assays) mentioned above, is the absence of pyoverdine production, a pigment distinctive of the fluorescent *Pseudomonas* group. Pyoverdine, major yellow-green Fe(III) chelating siderophore, has been reported as an important virulence factor of various species of *Pseudomonas*; even its function in the pathogenesis in some biological models of infection has been demonstrated [51,52]. To evaluate the pyoverdine levels present in the *oxyR-mutant* cultures, we carried out quantitative analysis of this siderophore at 28 °C and 18 °C. The results showed lower amounts of pyoverdine in the *oxyR-mutant* compared to the wt strain (*p* < 0.05) at both temperatures tested (28 °C and 18 °C). At 28 °C, the amount of pyoverdine in the culture supernatants of the wt strain was higher (21.42 ± 0.65) compared to the *oxyR-mutant* (1.49 ± 0.06). Likewise, higher pyoverdine levels in supernatants of the wt strain grown at 18 °C (17.17 ± 0.52) were observed compared to the *oxyR-mutant* (2.26 ± 0.78). No significant difference was observed between the cell culture supernatants grown at 28 °C and 18 °C for the *oxyR-mutant* (Figure 4). These findings were confirmed by observing the cell-free supernatants under exposure to UV-light (Figure 3). Additionally, the analysis of the supernatants of the *oxyR- C* strain showed a significant restoration of the pyoverdine levels at both temperatures compared to the *oxyR-mutant* (Figure 4). Thus, the results demonstrate that the synthesis of pyoverdine is a function of the OxyR protein.

To assess whether the low levels of pyoverdine observed in the *oxyR-mutant* strain are because of changes in the expression of the genes regulating the pyoverdine-synthesis, RT-PCR assays were performed evaluating the expression of the *pvdS* regulator gene. The PvdS alternative sigma factor protein plays an important role in the expression of all the genes of pyoverdine synthesis [53]. The results showed the absence of transcripts signals in the *oxyR-mutant* background at both temperatures evaluated compared to the wt strain (Figure 3). These results demonstrate that OxyR positively influences the expression of pyoverdine genes in *P. savastanoi* pv. *phaseolicola* NPS3121. 

Additionally, RT-PCR assays were performed evaluating the expression of the *sodB* gene encoding for the dismutase superoxide antioxidant enzyme. Like those obtained for the rest of the evaluated genes, the expression of the *sodB* gene was inhibited in the *oxyR-mutant* at both temperatures (28 °C and 18 °C) (Figure 3). These results demonstrate that the loss-function OxyR in the mutant strain influences the oxidative stress response.

## 4. Discussion

A key element in the development of the halo blight disease by *P. savastanoi* pv. *phaseolicola* is the effective synthesis of diverse virulence factors, among which phaseolotoxin has been highlighted. Despite its importance, knowledge about the regulatory mechanisms or signal transduction pathways related to the synthesis of this compound is very scarce. Therefore, it is necessary to do more research on this topic to better understand the molecular basis involved in this process. Previous works suggested that the OxyR protein is a member of the signal transduction pathway involved in the thermoregulation of phaseolotoxin, without this being verified yet. In this work, we initiated a study on the role of OxyR in *P. savastanoi* pv. *phaseolicola* by focusing on the control of phaseolotoxin production and the virulence of the bacterium. This study demonstrates that OxyR positively influences the expression of genes controlling phaseolotoxin synthesis and pyoverdine production, both important virulence determinants. Additionally, the effect of OxyR on the growth rate of *P. savastanoi* pv. *phaseolicola* was also reported.

The bacterial growth curves of the wt and *oxyR-mutant* strains of *P. savastanoi* pv. *phaseolicola* NPS3121 showed that OxyR plays an important role in the optimal growth of the bacterium, mainly at low temperatures (18 °C) and in minimal media conditions. The analysis of the bacterial growth curves in MM9, at 28 °C and 18 °C, showing a clear diminishment in the growth rate in the *oxyR-mutant* compared to the wt strain at both temperatures, while for those in the rich KB medium, the lack of OxyR only influenced the growth rate of *P. savastanoi* pv. *phaseolicola* NPS3121 at low temperatures (18 °C). The diminished bacterial growth rate was partially restored with the presence of the *oxyR* gene in trans (*oxyR-C*). No significant change was observed among the wt and *oxyR-mutant*s in KB medium at 28 °C. The influence of the OxyR global regulator on the growth of some bacterial pathogens had been previously reported. A positive influence of OxyR in the growth of *Vibrio parahaemolyticus*, determined by analysis of an *oxyR-mutant* strain, has been demonstrated [54]. Likewise, in *Shewanella oneidensis*, the *oxyR* mutation was implicated in the cell’s deprived ability to proliferate [55]. Conversely, the null influence of OxyR on the growth of *P. syringae* pv. *tomato* DC3000 has been also demonstrated. The growth curve assays carried out for the wt and *oxyR-mutant*s of *P. syringae* pv. *tomato* DC3000 in King´s B (KB) media showed that there was no significant difference between the bacterial growth among them [31]. These results are like those obtained in this study where the wt and *oxyR-mutant*s of *P. savastanoi* pv. *phaseolicola* NPS3121 grown in KB medium, showed similar growth dynamics at 28 °C. Some studies have demonstrated the influence of growth media composition and/or culture conditions on OxyR in bacterial growth [31,54]. Mostly, under conditions of rich media and/or shaken cultures, no change in the growth among the wt and *oxyR-mutant* strains has been observed in some pathogenic bacteria [31,54]. Based on this latter, the differences observed in this work in the growth rates of the wt and *oxyR-mutant* strains of *P. savastanoi* pv. *phaseolicola* NPS3121 grown in MM9 compared to those grown in rich KB medium could be due to the differences of composition of these media. Additionally, the analysis of the bacterial growth curves showed a negative effect of the low temperatures (18 °C) on the growth rate of both evaluated strains (wt and *oxyR-mutant*s). This effect of low temperatures had already been previously observed in several *P. syringae* strains and in *P. savastanoi* pv. *phaseolicola* NPS3121 [22,56]. However, the fact that the lack of OxyR resulted in implications in the growth rate mainly at 18 °C in a manner independent of the media composition used, suggests an important role of the OxyR protein in the physiology of the bacterium under low temperature conditions. Additional work is necessary to evaluate the way in which OxyR is involved in the growth of *P. savastanoi* pv. *phaseolicola* NPS3121, particularly at low temperatures.

The analyses concerning the influence of OxyR on the virulence and pathogenicity of the bacterium *P. savastanoi* pv. *phaseolicola* NPS3121 performed by bean pod assays, demonstrated that OxyR has a critical role in the virulence of the bacteria, but no roles in the pathogenicity processes. The in vitro phaseolotoxin assays confirmed these findings. The absence of the formation of halos of growth inhibition observed in the *oxyR-mutant* assays compared to the wt strain, and the restoration of these halos of growth inhibition in the *oxyR- C* strain, clearly demonstrate the implication of the *oxyR* gene in the ability of cells to produce phaseolotoxin, a major virulence factor of *P. savastanoi* pv. *phaseolicola* NPS3121. Similar findings have been previously reported in studies of various phytopathogenic bacteria. The positive influence of OxyR on the virulence of *P. syringae* pv. *tomato* DC3000 in Tomato and *Arabidopsis* plants has been demonstrated [31]. Likewise, OxyR is required for the virulence of the *Pantoea stewartii* and *Ralstonia solanacearum* bacteria [32,33]. The way in which OxyR is involved in the production of phaseolotoxin was determined by RT-PCR assays to evaluate the expression pattern of the genes of the Pht and Pbo clusters. The absence of transcript signals for the genes of both the Pht and Pbo clusters observed in the *oxyR-mutant* background, grown at 28 °C and 18 °C, demonstrate that the influence of OxyR on phaseolotoxin production is through the control or regulation of the expression of genes related to the synthesis of this compound. These results suggest that the OxyR protein acts as an activator to the expression of the phaseolotoxin genes. However, more experimental work is necessary to corroborate this. Although OxyR is primarily thought of as a transcriptional activator, it can function as either a repressor or activator under both oxidizing and reducing conditions [30,57]. On the other hand, despite that the expression of the *pboJ* gene in the wt strain was constitutive at both 28 °C and 18 °C, unlike the low-temperature-dependent expression of the remaining genes in the Pbo cluster and the Pht cluster genes, the *pboJ* expression was also decreased in the *oxyR-mutant* at both evaluated temperatures. These results demonstrate that OxyR influences the expression of the phaseolotoxin genes in a manner independent of the temperature. Furthermore, these results demonstrate that synthesis of phaseolotoxin and the genes of the Pht and Pbo clusters are members of the OxyR regulon in *P. savastanoi* pv. *phaseolicola* NPS3121.

The positive influence of OxyR on the expression of genes related to the virulence factors production has been documented in *P. syringae* pv. *tomato* DC3000, where OxyR promotes the expression of the *algD* gene involved in the exopolysaccharide alginate synthesis [31]. Similarly, OxyR positively influences the expression of various genes related to virulence in *P. aeruginosa* PAO1 [29]. On the other hand, and contrary to what was observed in this study, it has been reported that during infection of *P. syringae* pv. *tomato* DC3000, whose *oxyR-mutant* is compromised in its virulence to *Arabidopsis* and tomato plants, there is no direct implication of OxyR on the expression of genes related to the production of coronatine (*corR*), the main phytotoxin produced by this pathovar [31]. This agrees with what has been reported in that the targets of the OxyR protein are different, or particular, among bacteria [30].

A distinctive feature of the cultures of the *oxyR-mutant* of *P. savastanoi* pv. *phaseolicola* NPS3121 was the absence of the yellow-green pigment characteristic of the fluorescent *Pseudomonas* group, which is related to the pyoverdine siderophore production. The confirmation that OxyR influences the production of pyoverdine was performed by quantitative analysis of this siderophore. These analyses showed the presence of low levels of pyoverdine in assays with the *oxyR-mutant* strain compared to the wt strain. Pyoverdine levels in the *oxyR-mutant* were significantly restored by the presence in trans of the *oxyR* gene (*oxyR- C* strain). Through RT-PCR assays, we demonstrated that these low levels of pyoverdine are because of the decrease in the expression of the gene encoding to PvdS obtained in the *oxyR-mutant* strain at both evaluated temperatures. The PvdS sigma factor activates the transcription of pyoverdine biosynthethic genes [51]. Furthermore, the influence of PvdS in the expression of genes that encode virulence factors has been documented [51,53]. These results demonstrate that OxyR positively influences the pyoverdine synthesis by the positive regulation of the *pvdS* gene in *P. savastanoi* pv. *phaseolicola* NPS3121. The regulation of the *pvdS* gene by the OxyR protein had been previously reported in *P. aeruginosa* PAO1 [29]. On the other hand, the results of these analyses further demonstrate that pyoverdine synthesis and genes related to this process are also members of the OxyR regulon in *P. savastanoi* pv. *phaseolicola* NPS3121. 

The pyoverdine siderophore has also been reported to be an important virulence factor in some pathovars of *P. syringae,* implicated in the phytotoxins synthesis, extracellular polysaccharide, and the quorum sensing mechanism [58]. Therefore, the reduced virulence observed on the bean pod inoculation tests with the *oxyR-mutant* of *P. savastanoi* pv. *phaseolicola* NPS3121 could also be considered related to the low levels of pyoverdine present in this strain and not just with the inhibition of the phaseolotoxin synthesis. However, previous work in *P. syringae* pv. *tomato* DC3000 and *P. syringae* pv. *phaseolicola* 1448A have demonstrated that pyoverdine does not have a determinative role in the pathogenesis or virulence of these bacteria in their host plants [59].

The OxyR regulon has been widely studied in the *E. coli* and in *P. aeruginosa* bacteria. Although there are significant differences, the OxyR regulons of these and other microorganisms tend to include similar classes of genes as those involved in the defence to oxidative stress and iron homeostasis [29,30,60]. In particular, in *P. aeruginosa* PAO1, the OxyR regulon also includes genes involved in quorum-sensing, protein synthesis, oxidative phosphorylation, secretion and virulence factors encoding genes [29]. Thus far, the OxyR regulon of the *P. syringae* bacteria, including pv. *phaseolicola* has been poorly studied. Recent studies on *P. syringae* pv. *tomato* DC3000 have identified genes encoding thrioredoxins, thioredoxin reductase, and catalases, as being influenced by the OxyR protein. Likewise, genes involved in the alginate biosynthesis were identified as members of this regulon. The partial or indirect influence of OxyR on the expression of regulators of the T3SS and coronatine synthesis also was demonstrated [31]. Our study is the first report on members of the OxyR regulon in the *P. savastanoi* pv. *phaseolicola* NPS3121 bacterium.

## 5. Conclusions

In this study, we report that OxyR positively influences the synthesis of phaseolotoxin by regulation of the expression of genes of the Pht and Pbo clusters leading to a non-toxigenic phenotype of *P. savastanoi* pv. *phaseolicola* NPS3121. Likewise, the implication of OxyR in the synthesis of pyoverdine and oxidative stress is demonstrated in this study. 

## Figures and Tables

**Table 1:** The PCR primers used in this study.

Gene/Primers	Sequence (5´-3´)	Reference
**Pht Cluster**		
***argK***		
L10001	CTTTGATGGTATGCATGCGGTT	[12]
L10002	GGAAGAACTGGCCAAACATTCG	
***phtA***		
phtA fw	ATACTTTCCCTGTTTCCGGA	[23]
phtA rv	TAAACAGTGGTCAGCTTGTC	
***desI***		
P16881	TCAACAACATCCACGGGCAT	[12]
G720	GATATCGCAGCAACACCCATAAAAC	
***phtL***		
phtL fw	CTGGATGCATCTGTCGGAAT	[23]
phtL rv	GCCAGCAATGCATCGCTATG	
***amtA***		
BRL519	TTCATTCAAACCTCGCCCGTGTG	[12]
BRL520	TGAAAGGAGCCGCCGAAACTATTG	
**Pbo Cluster**		
***pboO***		
S126d	GCCGTTGTGATAGCCGACAGTGA	[15]
S127c	AACGCCAGCGCTTCATCCTTGT	
***pboL***		
S136d	CCACGCTGGACAACATGGTGATC	[15]
S137c	CATACTTTCTGGCCGCTACCCATTC	
***pboA***		
S134d	GCAAATTGCCAGTTGCGTTGCC	[15]
S135c	CCTTTCGGTGTACCGGTAGAACCAG	
***pboJ***		
S132d	TCTGTTCTGCAGCCTCAACGTGG	[15]
S133c	TGAGCTGGACAAATTCAATGGAGTGA	
**Pyoverdine**		
***pvdS***		
L100277-1909D	GACTCACCATTACTCCAGGC	[22]
L100278-1909R	AGACGGTACATCTCGAACGC	
**Control**		
**16S ribosomal**		
27f-1775	AGAGTTTGATCMTGGCTCAG	[39]
1492R-1776	TACGGYTACCTTGTTACGACTT	
**Construction of *oxyR-mutant***		
OxyR-FWD	AGCAGGCTCAAGGTATTCGT	This study
OxyR-REV	GAAGCGACCATGTGCCGAAT	This study
**Construction of *oxyR-* complemented mutant (*oxyR- C*)**		
pOxyR250-*Sma*I	CCCGGGACTCGATTGCCAACAGTTCAGG	This study
OxyRRev-*Bam*HI	GGATCCCTAACTTGCGACTGTTTTCGGA	This study
